# Cardiovascular Effect of Physical Exercise on Primary Sjogren's Syndrome (pSS): Randomized Trial

**DOI:** 10.3389/fmed.2021.719592

**Published:** 2021-09-30

**Authors:** Ana Beatriz Andrêo Garcia, Luciana Paula Dardin, Paulo Alexandre Minali, Virginia Fernandes Moça Trevisani

**Affiliations:** ^1^Department of Medicine, Health-Based Evidence, Universidade Federal de São Paulo, São Paulo, Brazil; ^2^Universidade de Santo Amaro, São Paulo, Brazil

**Keywords:** Sjogren' s syndrome, quality of life, physical activity, metabolic syndrome, subclinical cardiovascular disease

## Abstract

**Objective:** To evaluate the effects of an exercise program on aerobic capacity, echocardiographic parameters, metabolic profile, quality of life and safety in patients with primary Sjogren's syndrome in a randomized trial.

**Methods:** 60 women with pSS were evaluated from the SF-36 Short-Form Health Survey (SF-36) and EULAR Sjögren's Syndrome Disease Activity Index (ESSDAI) questionnaires. The participants performed ergospirometry and echocardiography; blood samples were collected to evaluate the metabolic profile. Patients were randomly divided into 2 groups: a training group that participated in the supervised training program and a control group. All variables were analyzed at baseline and after 28 weeks for both groups and we performed an intention-to-treat analysis. The training program consisted of 16 weeks of resistance exercises and, after, the exercise became aerobic. Patients and coaches were not blinded, contrary to the evaluators of all examinations/procedures and data analysts. Statistical analysis included Wilcoxon's rank sum test, chi-square test, and ANOVA test. *P* values < 0.05 were considered to be statistically significant.

**Results:** The 2 groups were homogeneous at baseline. The training group showed a significant improvement in oxygen maximum volume (VO2max) and anaerobic threshold (AT). Comparison of the training group and control group after 28 weeks showed a significant difference relating to VO2max and in AT. We did not find statistically significant diference in echocardiographic parameters, metabolic profile and in questionnaires SF-36 and ESSDAI.

**Conclusions:** This study showed significant improvement in aerobic capacity and glycated hemoglobin after a supervised training program in patients with pSS with safety.

## Introduction

Primary Sjogren's syndrome (pSS) is an autoimmune disease characterized by exocrine glandular dysfunction secondary to a local inflammatory reaction (1). It predominantly affects women between the fifth and seventh decades of life, with an estimated prevalence of 0.04–0.06% worldwide (2). Since the salivary and lacrimal glands are the main sites of lymphocyte infiltration, they account for the highest prevalence of symptoms associated with pSS, such as dry mouth and eyes (98 and 93%, respectively) (3). However, approximately one-third of pSS patients experience extra-glandular symptoms (4).

Recent studies have shown an increased cardiovascular risk (CVR) in patients with pSS. Bartoloni et al. reported that the prevalence of coronary and cerebrovascular diseases was higher in 788 female patients with pSS than in 4,774 healthy women (5). Zoller et al. followed patients with autoimmune diseases from 1987 to 2008 and found that the risk of acute myocardial infarction in patients with pSS was twice as high as that of the general population (6). A higher frequency of traditional risk factors, such as systemic arterial hypertension, diabetes mellitus (DM), and dyslipidemia (7, 8) in patients with pSS cannot explain the increased CVR observed, indicating that pSS is an independent risk factor for cardiovascular disease (6, 9).

Although the data are still scarce and inconsistent, some studies report an association between disease characteristics and increased CVR. For example, the presence of Ro/SSA autoantibodies has been shown to be associated with a diagnosis of subclinical atherosclerosis (10, 11), however, other studies have not been able to corroborate these results (5, 11). Clinical and laboratory data have also shown that parotitis and leukopenia may be associated with endothelial dysfunction and subclinical cardiovascular injury; however, to date, no other studies have confirmed these findings (12, 13).

More than 50% of pSS patients experience intense and incapacitating fatigue (14), which in turn promotes a sedentary lifestyle and increases CVR (15). Ng et al. found that the physical activity levels were 50% lower in individuals with pSS, as well as a marked reduction in moderate-to high intensity activities when compared to a control group (16). Strombeck et al. compared the aerobic capacity between pSS patients and healthy control subjects, reporting a reduction of ~11% in the maximal oxygen consumption (VO2max or VO2peak) of the pSS group and demonstrating that cardiac function is associated with pSS (17).

Physical exercise is one of the pillars of prevention for cardiovascular events. In addition to controlling metabolic risk factors, it promotes direct cardioprotection during ischemia-reperfusion, thereby attenuating tissue death and allowing greater maintenance of cardiac function. Although the mechanisms underlying this protective response are not yet clear, it is believed that nitric oxide metabolism, antioxidant systems, and cardiac opioids are involved in the process (18).

Thus, the objective of our study was to evaluate the impact of supervised physical exercise on aerobic capacity, echocardiographic parameters, metabolic profiles, quality of life, and disease activity in patients with pSS.

## Materials and Methods

This study was approved by the Ethics Research Committee of the Federal University of São Paulo (Unifesp) (opinion 503606/2013) and registered in the Brazilian Registry of Clinical Trials (Trial RBR-3V3ZMD). All the patients signed a free and informed consent (FIC), and all the principles outlined in the Declaration of Helsinki were followed.

### Eligibility Criteria

For the study, we recruited female patients between the ages of 18 and 90 years with an established diagnosis of pSS according to the 2002 American-European Consensus Group (4). The exclusion criteria included the diagnosis of secondary Sjogren's syndrome, history of cardiac and/or pulmonary diseases, an articular inflammation that prevents physical exercise, participation in a regular physical exercise regimen for more than 4 weeks in the 6 months prior to recruitment, and pregnancy.

The clinical, laboratory, and imaging examinations were performed at facilities located at the Hospital São Paulo and the Center for Studies in Psychobiology and Exercises (in Portuguese, Centro de Estudos em Psicobiologia e Exercícios—CEPE).

The patients who agreed to participate in the study and met the eligibility criteria were divided into two groups: the physical exercise group (EG) and the control group (CG). In the CG, patients followed the standard pSS visit and treatment protocol without additional intervention.

### Randomization and Blinding

Prospective study participants were invited to participate in the study by the principal investigator. After the eligibility assessment, the names of the included patients were given to an individual blinded to study who was responsible for the random generation of the allocation sequence and distribution of the intervention envelopes to the participants. The blinded randomization of patients into the EG or CG groups was conducted in a 1:1 ratio using Microsoft Excel (Microsoft Corp., Redmond, VA, USA). The volunteers received opaque, closed envelopes during their first visit, and opened by the patients in the presence of the coach on the second visit. Unlike examination/procedure evaluators and data analysts, the patients and coaches were not blinded to the study.

### Intervention

The groups were analyzed at baseline (T0) and after 28 weeks (T28). Under the guidance and supervision of a physiotherapist or physical education professional, the training was carried out in groups of five patients at the CEPE premises twice a week.

The program was divided into two phases. The first phase consisted of 16 weeks of resistance exercises in a 45-min circuit, and each muscle group was exercised for three sets of 12 repetitions each. In the first training session, we tested the maximal number of repetitions for each muscle group with a gradual increase technique to determine the maximum voluntary contraction (MVC), which consists of the heaviest weight the subject can shift during a complete movement without compensation. The training was individually designed to ensure that each patient performed the repetitions with 80% of the MVC. A new MVC assessment was performed every 2 weeks to make necessary adjustments. The circuit included the extensor chair (quadriceps), inclined leg press (quadriceps, hamstring muscles, gluteal muscles), horizontal leg press (calf muscles), abductor and adductor chair (abductor and adductor muscles, respectively), direct threading (biceps), French press (triceps), lateral elevation of the upper limbs (shoulder), crucifix (pectoral muscles), and bent-over row (grand dorsal muscle).

The second phase began after week 17, at which time the patients were switched to an aerobic exercise regimen while using an ergometer (the same one used in the ergospirometric test) and an electromagnetic braking cycle (Lode Excalibur Sport, Groningen, The Netherlands) coupled to a computerized gas analyzer (Quark CPET, Cosmed, Italy). Throughout phase 2, the training intensity and duration progressively increased. At weeks 17 and 18, the load was 20 to 39% of the VO2 max for 20 min; at weeks 19, 20, and 21, the load was increased to 40 to 59% of the VO2 max for 30 min. The training duration was then increased, while maintaining the same load, to 40 min and 50 min between weeks 22 and 24 and a load of 60 to 84% of the VO2 max in the last 3 weeks of the study. The ergometric test at T0 provided data for the maximum oxygen consumption. After making the appropriate percentage calculations, the intensity (in watts) corresponding to each incremental range to be used on the bike was established. The patients were instructed to maintain their exercise rate at between 60 and 70 rotations per min (rpm) and perform a 5-min warm-up on the bike before officially starting the workout.

Subjects in the EG who did not exercise at least 75% of the planned study time, or who were absent from more than two consecutive training sessions, were excluded.

### Outcome Measures

In a previous study, an average improvement of 12,5% in aerobic capacity was observed after performing aerobic exercise (19). Therefore, the primary endpoint of this study was to assess improvements of at least 15% in the patients' aerobic capacity compared to the baseline. This was accomplished by measuring the VO2 max using ergospirometry. Secondary endpoints included an intragroup assessment of the aerobic threshold (AT) and maximal heart rate through ergospirometry, echocardiographic parameters, metabolic profile, quality of life, and safety.

### Variable Analysis

Ergospirometry: patients were instructed not to consume stimulant foods or perform intense physical activity in the 24 h prior to the first visit. They were also told to wear comfortable clothes and sneakers and only eat light meals up to 1 h before the examination.

After a 3-min analysis of the gases, a progressive test was started, with an intensity increase of 10 watts every minute until the subject reached the maximum voluntary exhaustion, and the patients were reminded to maintain a pedaling frequency between 60 and 70 rpm. When the maximum effort was reached, the test was interrupted, and the patient was encouraged to maintain a low speed for two min to recover and an additional minute to record the vital signs and gas data. VO2 max and AT, expressed in mL/kg/min, were calculated using a gas analyzer. The maximal heart rate, measured concomitantly with the VO2 max, was also recorded.

Clinical evaluation, metabolic profile, and questionnaires: The patients were instructed to observe a 12-h fast. They underwent a medical evaluation to collect clinical and physical examination data and completed the validated Portuguese version of the Short-Form Health Survey SF-36 (20) and the EULAR Sjogren's Syndrome Disease Activity Index (ESSDAI) (21).

Peripheral blood was collected from the patients to evaluate disease activity, glycemic metabolism (fasting glycemia and glycated hemoglobin—HbA1c), lipidemic profile (total cholesterol, HDL and LDL fractions, and triglycerides), and the presence of autoantibodies. Urine samples were also collected.

Doppler echocardiography: a Doppler echocardiography was performed for each patient by an experienced cardiologist. Parasternal images were obtained along the longitudinal axis of the patients in the left lateral decubitus position. The dimensions of the left ventricle (LV) and thickness of the posterior and septal walls were measured using standard bidimensional echocardiography in M-mode, while the left ventricle ejection fraction (LVEF) was obtained according to the modified Simpson method. Left ventricular diastolic dysfunction (LVdd) was measured using the tissue Doppler according to the diastolic myocardial velocities obtained from the mitral ring in the septal and lateral basal segments of the LV by the apical cut of the four chambers. All the procedures were performed at T0 and T28 and at a maximum of 72 h after the last EG training session.

### Sample Size Estimate

A study performed by Strombeck et al. (17), in which they evaluated the aerobic capacity of pSS patients using the VO2 max, reported a mean of 28.7 mL/kg/min. Using a 90% test power and a significance level of 0.05, we estimated that 26 patients would be required to detect aerobic capacity improvements of at least 15% after the intervention.

### Statistical Analyses

We performed an intention-to-treat analysis of the patients who withdrew from the study after replicating the examination and questionnaire results from T0 to T28. Data are presented using descriptive statistics (mean ± standard deviation, absolute number, and percentage). The Shapiro-Wilk test was used to verify the hypothesis of a normal distribution of the data. The analyses were performed using the R program version 3.3.2 for Windows (R Foundation for Statistical Computing, Vienna, Austria), with a significance level of 5% (*p* < 0.05). The student's *t*-test was used for data with a normal distribution and the Wilcoxon test was used for data with a non-normal distribution. We used the Chi-square test to evaluate categorical variables and analysis of variance (ANOVA) to compare intergroup and intragroup variability. The effect of the intervention was verified using repeated-measures ANOVA.

## Results

The patients were recruited between January 2014 and June 2015 at the Hospital São Paulo/Unifesp, the Hospital do Servidor Público Estadual, and the Universidade de Santo Amaro, as well as through specific social network groups where the study was publicized (https://www.facebook.com).

**Figure 1:**
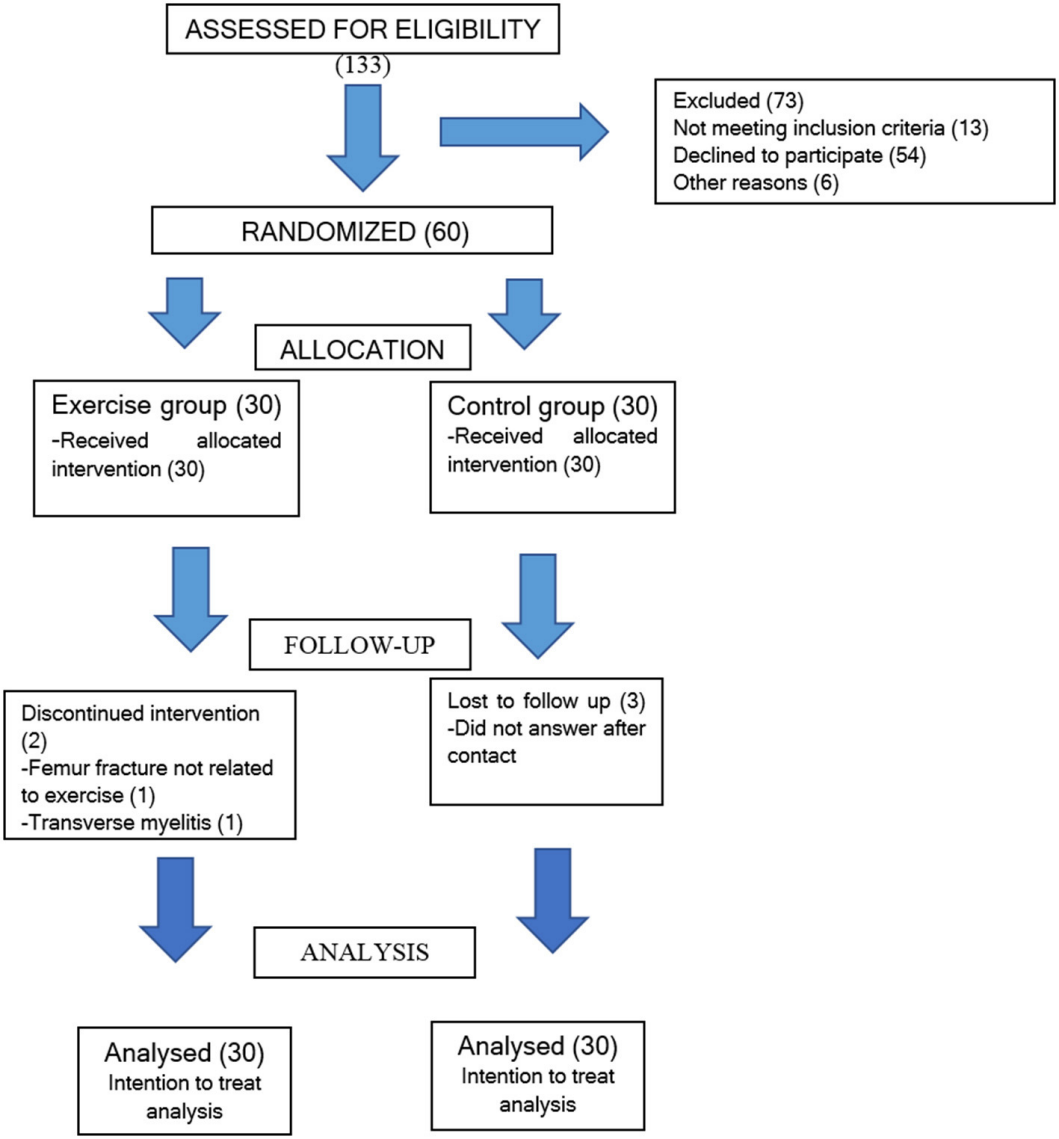
Flow diagram of participants through each stage of the trail.

The study was performed between February 2014 and February 2016. Patient recruitment is shown in Figure 1. Except for the ESSDAI, all data were normally distributed. Thus, the evolution of this variable was performed using Wilcoxon's non-parametric statistics.

Twenty-eight patients completed the exercise protocol, with average adherence 82% (22, 96 weeks), with minimum and maximum adherence of 78,57 (22 weeks) and 96,42% (27 weeks), respectively. Patient withdrawal from the study in the EG occurred at weeks 11 and 18, while all losses in the CG occurred at T28.

The baseline data of the groups were similar, except for the higher prevalence of antiSSB/La in the EG (17/30−56.7 vs. 8/30−26.7%, respectively, *p* = 0.018) and the greater use of corticosteroids in the CG (28/30−93.3 vs. 21/30−70%, respectively, *p* = 0.02) (Table 1).

**Table 1:** Clinical and demographic characteristics of the EG and CG groups at baseline.

	**Control (*****n*** **=** **30)**		**Exercise (*****n*** **=** **30)**		***p***
Age (years)	55.77	(10.42)	60.43	(12.20)	0.124
Disease d (years)	5.73	(4.55)	6.15	(4.20)	0.572
Symptoms d (years)	12.80	(8.62)	12.33	(7.27)	0.894
BMI	26.60	(4.11)	27.35	(4.20)	0.493
SAH	10	(33.3)	12	(40.0)	0.592
DM	5	(16.7)	3	(10.0)	0.448
DLP	5	(16.7)	11	(36.7)	0.080
Smoking					
Previous	4	(13.3)	4	(13.3)	0.601
Current	1	(3.3)	0	(0.0)	0.601
ESSDAI	3.43	(3.35)	2.47	(3.48)	0.151
VAS fatigue	6.50	(2.90)	6.93	(2.25)	0.736
ANF	22	(73.3)	23	(76.7)	0.766
RF	15	(50.0)	12	(40.0)	0.436
SSA/RO	17	(56.7)	19	(63.3)	0.598
SSB/LA	8	(26.7)	17	(56.7)	0.018
Diagnosis					
AP	13	(43.3)	10	(33.3)	0.598
Autoantibodies	17	(56.7)	19	(63.3)	0.598
Systemic Status	16	(53.3)	11	(36,7)	0.329
Drugs					
DMARD	13	(43.3)	11	(36.7)	0.268
Corticoid	28	(93.3)	21	(70.0)	0.020

*Values are mean and standard deviation for continuous variables and absolute number and percentage for categorical variables. Disease d, disease duration; Symptoms d, symptoms duration; BMI, body mass index; SAH, systemic arterial hypertension; DM, diabetes mellitus; DLP, dyslipidemia; ESSDAI, EULAR Sjogren's Syndrome Disease Activity Index; AVS, analog visual scale; ANF, antinuclear factor; RF, rheumatoid factor; SSA/Ro, antiSSA/Ro antibody; SSB/La, antiSSB/La antibody AP, anatomopathological; DMARD, disease-modifying antirheumatic drugs*.

### Ergoespirometry

There was a significant improvement in the VO2 max of the EG between T0 and T28, with a mean value of 17.95% (±14.85). Twenty patients (66,67%) had an increase of 15%. Among the patients who completed the exercise protocol, the minimum and the maximum increase were 3,82 and 53,45%, respectively.

After 28 weeks of supervised exercise, there was a significant increase in the VO2 max, (Δ +3,31 ml/kg/min−22.95 ± 4.01 vs. 19.64 ± 3.47, *p* < 0.001) and AT (Δ +2,7 ml/kg/min−19.56 ± 3.18 vs. 16.86 ± 2.86, *p* < 0.001) in the EG. In the CG, there was a significant decrease in the VO2 max (Δ −1,76 ml/kg/min−20.20 ± 4.16 vs. 21.96 ± 5.50, p=0.028) over the 28-week period, with no other changes to the other variables (Table 2).

**Table 2:** Assessment of the VO_2_ max, AT e HR max variables in the EG and CG groups before and after 28 weeks.

		**TIME POINTS**			
	**N**	**T0**	**T28**	**Δ**	***p***
VO_2_ max (ml/kg/min)					
CG	30	21,96 ± 5,5	20,2 ± 4,16	−1,76	0,028
EG	30	19,64 ± 3,47	22,95 ± 4,01	+3,31	< 0,001
*p* intergroup		0,056	0,012		
AT(ml/kg/min)						
CG	30	18,61 ± 3,67	17,8 ± 2,82	−0,81	0,163
EG	30	16,86 ± 2,86	19,56 ± 3,18	+2,7	< 0,001
*p* intergroup		0,065	0,041		
HRmax(bpm)					
CG	30	144 ± 18,3	139,9 ± 20,9	−4,1	0,106
EG	30	137,9 ± 16,3	142,1 ± 18,8	+4,2	0,106
*p* intergroup		0,405	0,405		

*VO_2_ max, maximum oxygen consumption; AT, aerobic threshold; HR max, maximum heart rate; bpm, beats per minute. P values provided by Anova method*.

**Figure 2:**
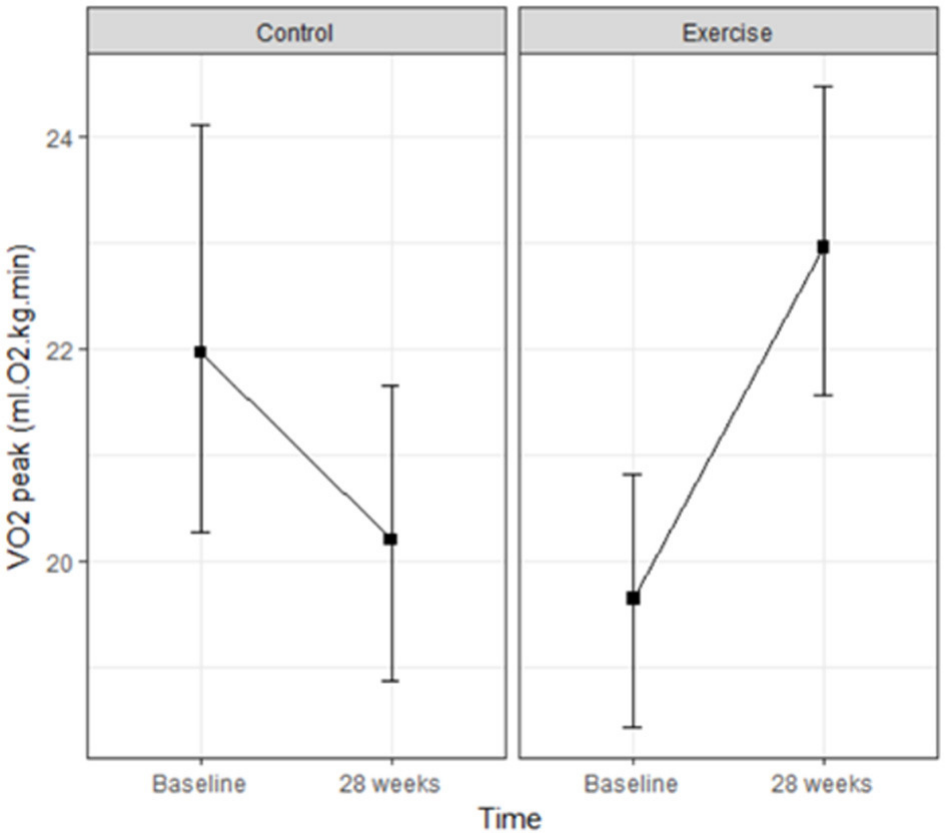
Mean VO2 peak values at each of the evaluation times in the control and exercise groups [F_(1, 58)_ = 31.43; *p* < 0.001].

In the intergroup evaluation, there was a significant improvement in the VO2 max [F_(1, 58)_ = 31.43; *p* < 0.001] and the AT [F_(1, 58)_ = 5.41; *p* < 0.001] in the EG compared to the CG. Figure 2 shows intragroup evaluation in CG and EG.

### Metabolic Profile, Doppler Echocardiography and Questionnaires

We found no intragroup differences in either group from baseline to the end of the study (T0 vs.T28). There were also no intergroup differences in any of these parameters.

We found a small, but significant, improvement in the HbA1c in the EG between T0 and T28 (Δ −0,13%−5.88 ± 0.73 vs. 5.75 ± 0.66, *p* = 0.006). The improvement observed in the intergroup evaluation was statistically significant in the EG compared to the CG [F_(1, 58)_ = 4.21; *p* = 0.001].

There were no significant differences between the groups at T28 in the intragroup evaluations of the LVEF and light LVdd. Similarly, no intergroup differences were observed between the groups.

The mean ESSDAI remained stable in the CG group. We also found no significant differences between the two groups at T28.

Both groups showed significant improvements in all the domains of the SF-36 questionnaire; however, the intergroup analysis showed no relationship with the intervention.

No harm or unintended effects occurred in either group.

## Discussion

Low aerobic capacity has been studied for more than two decades as an independent risk factor for cardiovascular mortality and overall mortality (22, 23). Low aerobic capacity can be objectively evaluated with an ergospirometry examination through the calculation of the VO2 max. Conversely, evidence shows that aerobic exercise helps in the treatment and control of cardiovascular risk factors such as hypertension, diabetes and obesity and can reduce the risk of cardiac events with efficacy similar to that of pharmacological treatments (24, 25).

Strombeck et al. were the first to demonstrate that women with pSS have a lower aerobic capacity than age-matched healthy controls (28.7 vs. 32.4 mL/kg/min) (16). In 2017, Dassouki et al. reported a lower aerobic capacity (22.5 ml/kg/min ± 3.5 vs. 24.6 ± 3.6), lower functionality, and higher levels of fatigue and osteoarticular pain in pSS patients than in those without pSS (26). Our study showed the aerobic capacity in pSS similar to Dassouki et al., with a mean value of 21.96 ml/kg/min ± 5.5 in the CG and 19.64 mL/kg/min ± 4.16 in the EG and with no significant differences between the groups.

We started our exercise program with 16 weeks of resistance training to increase muscular strength and functional capacity to improve aerobic training performance and decrease the occurrence of exercise-related injuries. After the 28-week program ended, we observed an increase of 17.95 ± 14.85% in the VO2 max of the EG, whereas the CG showed a significant decrease in this same parameter. We observed a significant improvement in the EG AT, considered a useful parameter for measuring training progress (27).

To date, only two studies have analyzed the effects of physical exercise on the aerobic capacity of patients with pSS. Strombeck et al. assessed nine patients for 12 weeks. Their program included walking three times a week, but only one of these sessions was supervised. The authors reported a significant improvement in the VO2max of these patients compared to the 10 healthy volunteers in the control group (19). In 2019, Miyamoto et al. followed 23 patients in a supervised walking program three times a week for 16 weeks and showed positive results in aerobic capacity compared to 22 participants in the control group (28).

Although data for other rheumatic diseases are still scarce, the number of studies in this field has been increasing. Ayan et al. recently published a systematic review evaluating the effects of physical exercise on patients with systemic lupus erythematosus (SLE). Of the 14 articles selected for their review, there were 13 studies involving adults with exercise programs ranging between 6 and 12 weeks. All the studies analyzed the impact of aerobic exercise, but only one combined this modality with resistance exercises. Five studies used aerobic capacity as a variable, and four observed a significant difference in the VO2max after the intervention. Thus, although the study's authors determined that the quality of the analyzed evidence was not high, the review showed that physical exercise can increase the aerobic capacity of patients with SLE (29).

Another systematic review evaluated 17 randomized controlled trials that analyzed the effects of exercise on patients with rheumatoid arthritis. Although the studies included differing exercise programs did not include descriptions of intervention monitoring, the authors concluded that medium-to high-intensity exercise can improve aerobic capacity and is therefore recommended as part of an overall treatment plan for patients with rheumatoid arthritis (30).

The effects of exercise on glycemic and lipidemic metabolism are widely known. Previous research has shown that exercise enhances glycemia and insulin sensitivity, which leads to better prevention and control of type 2 DM (31, 32). Exercise has also been shown to promote an increase in HDL levels and decreases in LDL, VLDL, and triglyceride levels (33).

In the present study, we did not observe any changes in the participants' cholesterol profiles, but there was a small, but significant improvement in the glycated hemoglobin levels. We believe that this benefit would be greater with increasing exercise frequency, as structured exercise training of more than 150 min per week is associated with greater declines in glycated hemoglobin compared to 150 min or less per week (34). To date, however, no studies demonstrated a link between exercise and the metabolic profile of patients with pSS.

Only one study has evaluated the lipidemic profile of SLE patients who underwent an aerobic exercise program twice a week for 12 weeks, but no improvements in the metabolic parameters measured were observed (35). This lack of change in cholesterol levels after exercise may be related to the presence of various pro-inflammatory cytokines, such as tumor necrosis factor-alpha and interferon gamma, as these proteins interfere with cholesterol synthesis and degradation (36). Previous studies have shown that these factors can reduce the activity of lipoprotein lipase, an enzyme associated with HDL synthesis (37).

Stavropoulos-Kalinoglou et al. conducted a 6-month intervention for RA patients, with aerobic exercises in the first 3 months and aerobic exercise in combination with resistance exercise until the end of the study. The patient HDL cholesterol levels underwent clinically significant changes by the end of the program, but there were no changes in the glycemic profile or total cholesterol and its components (38).

Echocardiographic analyses of pSS patients htipically show a silent impairment of the myocardium, mainly consisting of LV systolic and diastolic dysfunction. The mechanism underlying this dysfunction is still unknown, but it may be associated with Raynaud's phenomenon of coronary microcirculation or direct myocardial tissue injury caused by autoantibodies (39). Bayram et al. reported a higher prevalence of LV dysfunction in women with pSS (40). Similar results were reported by Vassiliou et al. and Manganelli et al. in pSS patients and Elnady et al. and Aslan et al. in patients with SLE and RA, respectively (40–43). In our study, 55.33% of the patients presented with LV diastolic dysfunction, with no changes in systolic function as measured by the ejection fraction.

Sarajlic et al. followed RA patients for 1 year enrolled in a moderate-to-intense unsupervised exercise program for 30 min on most days, with 2 weekly 45 min circuit sessions combining aerobic and resistance exercise. In the second year, the patients were encouraged to maintain their activity levels. There were no changes observed in the patients' LV systolic function, but there was an improvement in diastolic dysfunction seen after 1 year. The authors suggested that this is related to aerobic capacity, suggesting that impaired myocardial relaxation may be associated with a low VO2max (44). Although physical exercise promotes cardiac remodeling and thus improves ejection fraction and diastolic dysfunction, we did not observe any changes in the echocardiographic parameters evaluated in our study, probably due to shorter duration of the protocol.

As noted in the Miyamoto et al. study, the patients in our study did not experience worsened disease activity, confirming the safety of the exercise program (28). Similar results were observed in previous studies of patients living with SLE and RA (29, 30).

In our study, the patients presented low quality of life scores according to the SF-36 questionnaire, which aligns with other studies (45). Both groups improved in all the domains tested; however, similar to the results reported by Strombeck et al. and Miyamoto et al., we did not observe a significant difference between the two groups by the end of the program (27, 28). We agree with Miyamoto et al. that the number of meetings between CG and health professionals may have been a factor in raising mood, motivation and effort, which may influence the results for this group. Studies on quality of life for individuals with SLE have produced controversial results. Seven studies have used the SF-36 questionnaire for assessments after physical exercise for this population. In three, there were no improvements in any of the observed parameters, whereas the other studies reported significant improvements in up to three domains (29).

Our study had some limitations. This was a small, single-center study. In a larger sample, differences at baseline may be more defined. The recommendation to prescribe physical exercise for patients with pSS follows the standard prescription of exercise for healthy individuals in terms of intensity, duration, and frequency (46). In our study, however, the exercise program we designed followed the first two parameters, but the frequency was below the recommended minimum, (three times a week) because of the availability of volunteers (47). Even so, it was possible to promote the improvement of aerobic capacity, which has already been seen in patients with limited cutaneous Systemic Sclerosis (48).

In conclusion, ours results show that regular physical exercise based on personalized and supervised training can safely increase the aerobic capacity of patients with pSS.

## Data Availability Statement

The raw data supporting the conclusions of this article will be made available by the authors, without undue reservation.

## Ethics Statement

The studies involving human participants were reviewed and approved by Ethics Research Committee of the Federal University of São Paulo (Unifesp) (opinion 503606/2013). The patients/participants provided their written informed consent to participate in this study.

## Author Contributions

All authors listed have made a substantial, direct and intellectual contribution to the work, and approved it for publication.

## Funding

This work was supported by the Universidade Federal de São Paulo.

## Conflict of Interest

The authors declare that the research was conducted in the absence of any commercial or financial relationships that could be construed as a potential conflict of interest.

## Publisher's Note

All claims expressed in this article are solely those of the authors and do not necessarily represent those of their affiliated organizations, or those of the publisher, the editors and the reviewers. Any product that may be evaluated in this article, or claim that may be made by its manufacturer, is not guaranteed or endorsed by the publisher.
